# Application of HPCCC Combined with Polymeric Resins and HPLC for the Separation of Cyclic Lipopeptides Muscotoxins A–C and Their Antimicrobial Activity

**DOI:** 10.3390/molecules23102653

**Published:** 2018-10-16

**Authors:** José Cheel, Jan Hájek, Marek Kuzma, Kumar Saurav, Iva Smýkalová, Eliška Ondráčková, Petra Urajová, Dai Long Vu, Karine Faure, Jiří Kopecký, Pavel Hrouzek

**Affiliations:** 1 Laboratory of Algal Biotechnology-Centre ALGATECH, Institute of Microbiology of the Czech Academy of Sciences, Opatovický mlýn, Novohradská 237, 379 81 Třeboň, Czech Republic; hajek@alga.cz (J.H.); sauravverma17@gmail.com (K.S.); urajova@alga.cz (P.U.); longvu@alga.cz (D.L.V.); kopecky@alga.cz (J.K.); 2 Laboratory of Molecular Structure Characterization, Institute of Microbiology of the Czech Academy of Sciences, Vídeňská 1083, 142 20 Prague, Czech Republic; kuzma@biomed.cas.cz; 3 Plant Biotechnology Department, AGRITEC Plant Research Ltd., Zemědělská 2520/16, 787 01 Šumperk, Czech Republic; smykalova@agritec.cz (I.S.); ondrackova@agritec.cz (E.O.); 4 Institut des Sciences Analytiques, University of Lyon, CNRS, Université Claude Bernard Lyon 1, Ens de Lyon, UMR 5280, 5 rue de la Doua, 69100 Villeurbanne, France; karine.faure@isa-lyon.fr

**Keywords:** cyanobacteria, cyclic lipopeptides (CLPs), muscotoxins, high performance countercurrent chromatography (HPCCC), antimicrobial activity, γ-methylproline

## Abstract

Muscotoxins are cyanobacterial cyclic lipopeptides with potential applications in biomedicine and biotechnology. In this study, *Desmonostoc muscorum* CCALA125 strain extracts were enriched by polymeric resin treatment, and subjected to HPCCC affording three cyclic lipopeptides (**1**–**3**), which were further repurified by semi-preparative HPLC, affording **1**, **2**, and **3**, with a purity of 86%, 92%, and 90%, respectively. The chemical identities of **2**–**3** were determined as muscotoxins A and B, respectively, by comparison with previously reported ESI-HRMS/MS data, whereas **1** was determined as a novel muscotoxin variant (muscotoxin C) using NMR and ESI-HRMS/MS data. Owing to the high yield (50 mg), compound **2** was broadly screened for its antimicrobial potential exhibiting a strong antifungal activity against *Alternaria alternata*, *Monographella cucumerina*, and *Aspergillus fumigatus*, with minimum inhibitory concentration (MIC) values of 0.58, 2.34, and 2.34 µg/mL; respectively, and weak antibacterial activity against *Bacillus subtilis* with a MIC value of 37.5 µg/mL. Compounds **1** and **3** were tested only against the plant pathogenic fungus *Sclerotinia sclerotiorum* due to their low yield, displaying a moderate antifungal activity. The developed chromatographic method proved to be an efficient tool for obtaining muscotoxins with potent antifungal properties.

## 1. Introduction

Cyanobacteria are Gram-negative photosynthetic microorganisms that have gained attention as a source of potential therapeutically useful compounds [1]. One group of these compounds is represented by cyclic lipopeptides (CLPs), which have been found to comprise up to 14 amino acid residues, together with fatty acid chains that vary in length. CLPs are biosynthesized by multifunctional protein complexes called polyketide synthases (PKS) and non-ribosomal peptide synthetases (NRPS) [2]. In these structures, a modified fatty acid tail is linked to the peptidic macrocycle leading to the formation of CLPs. The pharmacological potential of CLPs is represented by their antifungal [3], antibiotic [4], cytotoxic [5], and antiproliferative activities [6,7]. Caspofungin, cyclosporine A, and daptomycin are three successful examples of CLPs that have been approved for clinical use as antifungal [8], immunosuppressant [9], and antibiotic agents in humans [10], respectively. Pharmacodynamically, CPLs and cyclic microbial peptides, in general, offer advantages as compared to the open forms because a cyclized peptide is less flexible and, therefore, more pre-organized, thus exerting a better affinity to their biological target due to the reduced entropy needed for target–ligand interaction [11,12]. In addition, CLPs are less sensitive to proteolytic degradation, thereby favoring their stability and bioavailability [12].

Muscotoxins are cyanobacterial CLPs comprising 11 amino acid units including a *β*-amino fatty acid [13]. Our group reported the separation and identification of cyclic undeca-lipopeptides muscotoxins A and B, as a mixture, from the soil cyanobacterium *Desmonostoc muscorum* CCALA125 (synonymous to Lukešová 1986/14 and NIVA-CYA 817). The structures of these compounds were determined by detailed HRMS/MS and NMR analysis, and Marfey’s reagent was used for chiral amino acid analysis after acid hydrolysis [13]. Muscotoxins A and B were observed to differ in the substitution of proline by γ-methylproline. So far, the separation of muscotoxins from cyanobacterial biomass has only been performed by the application of multi-step procedures including the use of liquid–liquid partitioning and chromatographic methods of solid support, such as solid-phase extraction on cartridges and high-performance liquid chromatography (HPLC) [13]. However, these methods did not exert enough resolution capacity for separating muscotoxin A from its congener B, and did not achieve high yields of minor muscotoxins from cyanobacterial biomass. Hence, the development of an efficient and high-throughput separation method for obtaining these compounds from cyanobacterial biomass is warranted to enable their extensive in vitro and in vivo studies, and to efficiently support drug discovery strategies focused on CLPs of medical importance.

High-performance countercurrent chromatography (HPCCC) is a solid support-free chromatographic technique that uses two immiscible solvent phases. The equipment uses centrifugal force for retaining the stationary phase within the column, while the mobile phase is pumped through the column [14]. Those compounds with different partition coefficients between the two immiscible phases could be separated by HPCCC. Since the stationary phase in HPCCC is liquid, there is no risk of irreversible adsorption of target compounds, it is possible to load a high amount of sample, the technique permits a high recovery of analytes, and there is a low risk of sample denaturation [15]. Countercurrent chromatography has been successfully used in the isolation of several polyphenols, betalains, and flavonoids from *Cymbopogon citratus* [16], *Beta vulgaris* [17], and *Siparuna glycycarpa* [18], respectively. The isolation of the bioactive carotenoid zeaxanthin from the cyanobacteria *Microcystis aeruginosa* [19], and canthaxanthin from the microalga *Chlorella zofingiensis* [20], using countercurrent chromatography methods, have also been reported. However, the application of HPCCC for isolation of CLPs has not been explored extensively [21].

As muscotoxins belong to the class of cyclic lipopeptides usually possessing antimicrobial activities, they could be interesting targets for addressing combinatorial biosynthesis strategies aimed at enhancing their potential pharmacological efficacy or mitigating toxicity issues. Herein, we report the separation of muscotoxins from cyanobacteria by the combined use of HPCCC, polymeric resins, and HPLC. The isolated compounds were tested for their antimicrobial activity against a panel of fungi, as well as Gram-positive and Gram-negative bacteria.

## 2. Results and Discussion

### 2.1. HPLC-ESI-HRMS Analysis of the Crude Extract

**Figure 1:**
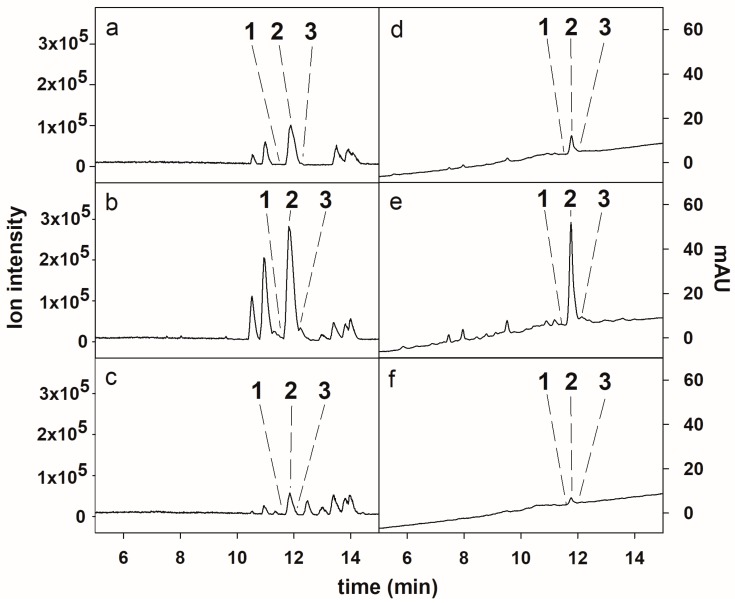
ESI-HRMS base peak (**a**–**c**) and UV (**d**–**f**) chromatograms of the crude extract from *D. muscorum* CCALA125 (**a**,**d**), the muscotoxin-enriched extract obtained by consecutive treatment on Amberlite XAD-16 (**b**,**e**) and Amberlite XAD-7 (**c**,**f**) adsorption resins. The UV chromatograms were monitored at 240 nm.

The chemical identities of the compounds **2** and **3** from *D. muscorum* CCALA 125 extract were determined by comparing their ESI-HRMS/MS spectra with the literature data [13] (see Section 2.6). Compound **2**, the major CLP in the extract (Figure 1a,d), was assigned to muscotoxin A. The compounds **1** and **3**, which were present at trace amounts in the crude extract, were identified as an unknown muscotoxin congener and muscotoxin B, respectively. In order to achieve the isolation of the minor target compounds, the enrichment of the crude extract was necessary prior to the isolation.

### 2.2. Enrichment of the Crude Extract by Adsorption Resins

**Figure 2:**
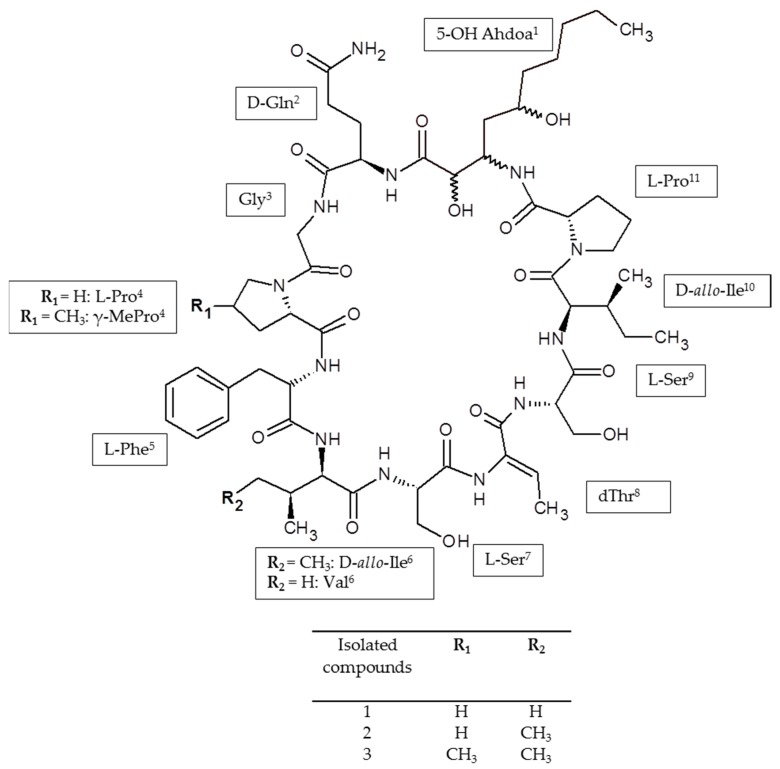
Chemical structures of muscotoxins isolated from the *D. muscorum* CCALA 125. Ahdoa^1^ (3-amino-2-hydroxydecanoic acid), d-Gln^2^ (d-glutamine), Gly^3^ (glycine), l-Pro^4,11^ (l-proline), γ-MePro^4^ (γ-methylproline), l-Phe^5^ (l-phenylalanine), d-allo-Ile^6,10^ (d-alloisoleucine), Val^6^ (valine), l-Ser^7,9^ (l-serine), dThr^8^ (dehydro-threonine).

The crude extract was enriched with the target compounds by consecutive adsorption on the non-ionic polymeric resins Amberlite XAD-16 and Amberlite XAD-7. Amberlite XAD-16 is made of a hydrophobic styrene-divinylbenzene matrix, while Amberlite XAD-7 is made of a moderately polar acrylic matrix. The application of these non-ionic polymeric resins, either using a single type of resin [22,23,24,25] or combining different resins [21,25], has shown great efficiency in the purification of cyclic peptides from cyanobacterial and bacterial extracts. In the current study, 6200 mg of crude extract were consecutively processed by Amberlite XAD-16 resin affording 700 mg of enriched extract, and 300 mg of enriched extract from Amberlite XAD-7 resin treatment. As was determined by HPLC-ESI-HRMS analysis (Figure 1b,c) the Amberlite XAD-16- and Amberlite XAD-7-enriched extracts were similar in their qualitative profiles, and they were pooled, affording 1000 mg of enriched extract for further HPCCC isolation The Amberlite XAD-16-enriched extract exhibited the highest peak purities of the target compounds (Figure 1b). The chemical structures of the target compounds are shown in Figure 2.

### 2.3. Optimization of the HPCCC Conditions

When working with HPCCC, the search for a proper two-phase solvent system has been informed to represent the major part of the work involved in the HPCCC separation [14]. An appropriate two-phase solvent system for HPCCC has to provide a good partition coefficient (0.5 ≤ *K* ≤ 2.5) for the target compound. Moreover, it should exhibit a short settling time (less than 30 s) and a suitable density difference between the upper and lower phases of the two-phase solvent system, in order to favor the retention of stationary phase inside the HPCCC column [14,26], which favors the chromatographic resolution. Additionally, for achieving a good resolution between two closely eluting peaks, the separation factor (α) between the two compounds (α = *K*_2_/*K*_1_, *K*_2_ > *K*_1_) should be greater than 1.5 [14,26]. It is well described that compounds with smaller *K* values show a poor resolution because they elute closer to the solvent front. On the contrary, those compounds with larger *K* value are better separated but elute as wider peaks and at later elution times [14]. In the present study, the capacity of different two-phase solvent systems (Table 1) to provide suitable *K* values of the compounds **1**–**3** was investigated. The *K* value is frequently estimated by using HPLC with UV detection, which is calculated by dividing the peak area of the target compound in the upper phase by that of the peak area of the same target compound in the lower phase. However, given that UV chromatogram provided insufficient peak areas for minor muscotoxins, the MS detector was thus used to estimate the *K* values. By using this approach, the peak area of the molecular ions corresponding to the target compounds were selectively monitored from the base peak chromatogram. As presented in the Table 1, the solvent system 10 (*n*-hexane, ethyl acetate, ethanol, and water in a ratio of 1:5:1:5) gave suitable *K* values and a proper separation factor (α) for compounds **2** and **3**, as well as a short settling time. However, the solvent system 10 gave a small *K* value (*K* ≤ 0.5) for compound **1**, indicating that this compound would be poorly separated from other contaminants. Hence, to have a suitable *K* value, the solubility of compound **1** in the upper phase should be increased. The improvement of the *K* value of compound **1** could be achieved by finding out a new solvent system or by modifying the composition of the already selected solvent system. In the present study, the system 10 was acidified by adding acetic acid leading to the system 11 (*n*-hexane, ethyl acetate, ethanol, water, and acetic acid, 1:5:1:5:1). This modification favored the solubility of compound **1** in the upper phase of the solvent system 11, giving rise to an appropriate partition coefficient (*K* = 1.09), a short settling time, and a suitable density difference. Although the partition coefficients of the target compounds in systems 10 and 11 are strongly correlated, the system 10 ensured a sufficient selectivity (α = 1.5) between the compounds **2** and **3**. Overall, these results indicated that the separation of the compounds **1**–**3** would demand the application of a two-step HPCCC method. In this context, the solvent system 10 showed selectivity for the compounds **2** and **3**, and solvent system 11 was selective for the compound **1**. In HPCCC, it is very well established that when the retention of stationary phase is increased, a good chromatographic resolution is achieved [27]. A reduced flow rate of the mobile phase and a high rotational speed of the column favor the retention of the stationary phase inside the HPCCC column. In this study, the flow rate of the mobile phase in the first step and second step of the HPCCC operations was 1 mL/min and the rotational speed was set at 1200 rpm. Moreover, it was observed that a good resolution between the compounds **2** and **3** was achieved when injecting 100 mg of enriched extract.

### 2.4. HPCCC Separation of Compounds ***1**–**3***

**Figure 3:**
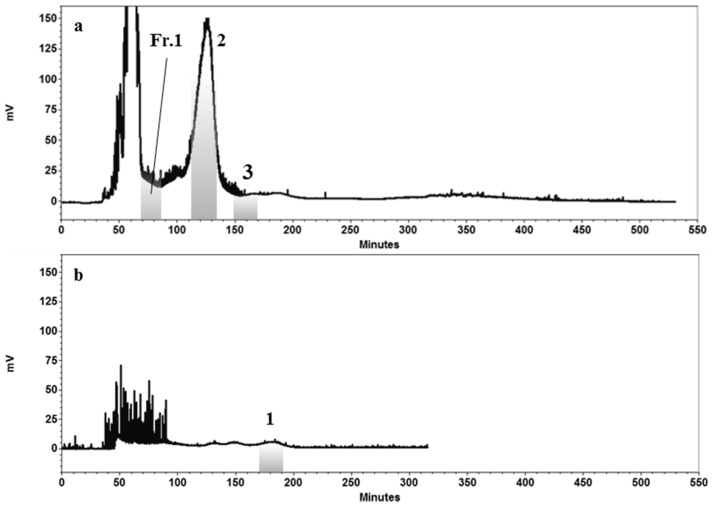
Chromatogram of (**a**) the first step HPCCC separation of amberlite enriched extract using solvent system 10, yielding **Fr.1**, **2**, and **3**, and (**b**) second step HPCCC separation of **Fr.1** using solvent system 11, yielding compound **1**.

The optimized operating conditions were used to process 1000 mg of enriched extract using HPCCC, where the lower phase of the solvent system 10 (*n*-Hex–EtOAc–EtOH–H_2_O, 1:5:1:5) was used as mobile phase at a flow rate of 1 mL/min. This process was repeated 10 times to separate all enriched extract. The stationary phase retention during the HPCCC separation was 72%. Three peaks fractions that were eluted at the retention times from 68 to 88 min, from 110 to 135 min, and from 150 to 170 min, corresponded to the compound **1**-containing fraction (**Fr.1**), compound **2**, and compound **3**, respectively (Figure 3a). The obtained fractions were concentrated using a rotatory evaporator leading to **Fr.1** (10%, HPLC purity), as well as the compounds **2** (92%, HPLC purity) and **3** (88%, HPLC purity). **Fr.1** was further subjected to a second HPCCC purification step using solvent system 11, with its lower phase as the mobile phase at a flow rate of 1 mL/min. The target compound **1** was eluted at the retention times from 170 to 190 min (Figure 3b), which was subsequently collected and concentrated, obtaining 3.5 mg of compound **1** (89%, HPLC purity). The stationary phase retention during the HPCCC separation was 70%. In this study, the isolation of compound **1** is reported for the first time. The separation of this kind of CLPs with closely related chemical structures demonstrates the powerful selectivity of HPCCC.

### 2.5. HPLC Purification of the Compounds Obtained from HPCCC

The combined use of HPCCC and HPLC has proven to be efficient in the isolation of CLPs [21]. This chromatographic complementarity is due to HPCCC being orthogonal to HPLC, which is explained by the fact that the separation of compounds in both the techniques is governed by different mechanisms. In the present study, the HPCCC peak fractions corresponding to compounds **1**–**3** were repurified by HPLC to increase their purity. Overall, the compounds **1** (0.95 mg, 86% HPLC purity), **2** (53.4 mg, 92% HPLC purity), and **3** (0.8 mg, 90% HPLC purity) were obtained by HPLC when considering the stereoisomers of the target compounds as contaminants (Figure S1). The HPLC-ESI-HRMS chromatograms of the isolated compounds are shown in Supplementary Materials (Figure S1).

### 2.6. Identification of the Target Compounds

**Figure 4:**
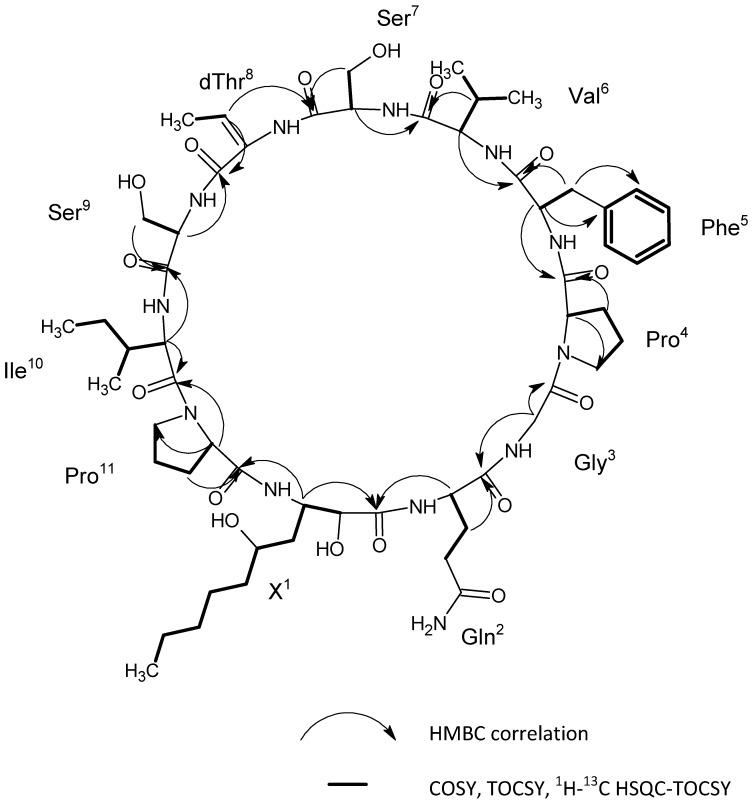
Selected correlations obtained by 2D NMR spectroscopy experiments for compound **1**.

**Figure 5:**
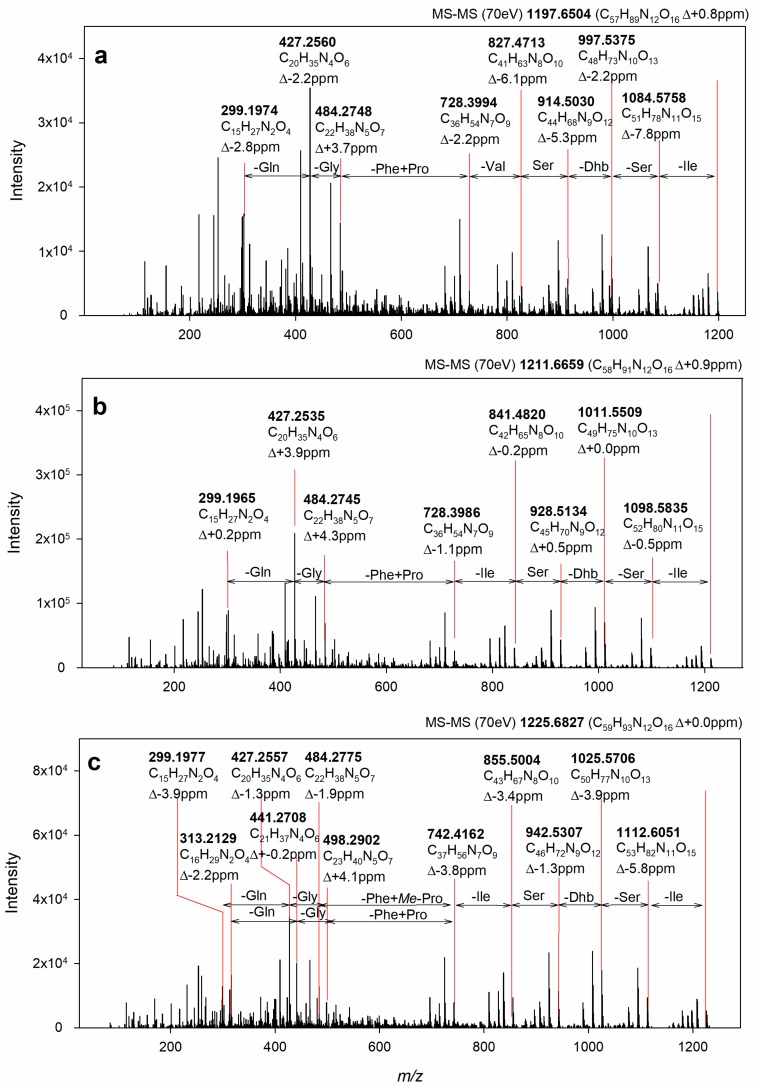
ESI-HRMS/MS spectra of isolated compounds **1** (**a**), **2** (**b**), and **3** (**c**). The HRMS/MS analysis confirmed the identity of the compounds **2** and **3** as muscotoxin A and muscotoxin B, respectively. Compound **1** was identified as novel muscotoxin variant (muscotoxin C) based on ESI-HRMS/MS (**c**) and NMR data. The sum formulas and mass error are shown for individual fragment peaks.

As demonstrated in Figure 4, the identity of **2** and **3** was confirmed by ESI-HRMS/MS experiments. The fragment ions generated from the fragmentation of the molecular ions at *m*/*z* 1211.67 (**2**) and *m*/*z* 1225.68 (**3**) were in excellent agreement with those of the amino acid sequence of muscotoxins A and B; respectively [13], that had been previously isolated from the strain *D. muscorum* CCALA 125. Compounds **2** and **3** differ from each other only by a -CH_3_ group (methyl group) corresponding to the substitution of l-proline for γ-methylproline, as previously demonstrated by our team using 2D NMR methods [13]. The chemical structure of **1** was determined by the combination of NMR and ESI-HRMS/MS. Two-dimensional nuclear magnetic resonance spectroscopy (2D NMR) experiments including COSY, TOCSY, and ^1^H-^13^C HSQC-TOCSY were performed to identify the amino acids composition of **1**, as shown in Supplementary Materials (Figure S2). The following amino acid residues were identified: Gln, Gly, dThr, Ile, Val, Phe, 2× Pro, and 2× Ser (Table 2, Figure 4). Furthermore, the non-amino acid residue, 2-hydroxy-3-amino-5-hydroxy decanoic acid (X residue) was identified based on the comparison of experimental data (Table 2, Figure 4) with the NMR data published previously [13]. The amino acid sequence was determined by HMBC using the correlations of the carbonyls to the Hα and Hβ (Figure 4). As a result, the obtained sequence was cyclo (X^1^, Gln^2^, Gly^3^, Pro^4^, Phe^5^, Val^6^, Ser^7^, dThr^8^, Ser^9^, Ile^10^, Pro^11^) (Figure 4). This peptide sequence was fully confirmed by the ESI-HRMS/MS fragmentation experiments (Figure 5). The novel muscotoxin variant (**1**) was assigned as muscotoxin C, which differs from the previously described muscotoxin A (**2**) in the substitution of Ile^6^ by Val^6^ (Figure 2).

### 2.7. In Vitro Antimicrobial Activity

The three isolated compounds (**1**–**3**) were tested for their antifungal activity against common plant pathogenic fungi, *S. sclerotiorum*. Interestingly, despite the isolated variants differing only in one amino acid residue, their antifungal activity against *S. sclerotiorum* differed significantly (Figure S3). The highest activity was observed for compound **3**, with 25 µg as the lowest dose to manifest its activity, followed by compound **2** exhibiting the inhibitory affect at 100 µg of dosage. Finally, compound **1** did not exhibit any significant activity even at the highest dosage used (250 µg). These data hint the possible role of the amino acid substitution in the peptidic core of the molecule, such as Pro by γ-MePro or Val by Ile enhancing the bioactivity. Owing to the high-yield isolation of compound **2** and the unavailability of sufficient amounts for minor variants isolated (**1** and **3**), the antimicrobial activity against five bacterial and eight fungal isolates was investigated only for compound **2**. Among all the bacterial strains tested, **2** showed a weak antibacterial activity only against the Gram-positive pathogen *Bacillus subtilis* with a minimum inhibitory concentration (MIC) value of 37.5 µg/mL and MIC_95_ at ≤300 µg/mL. However, it exhibited a moderate to strong antifungal activity against all the fungal strains tested, except *Bipolaris sorokiniana*. The strongest activity was observed against *Alternaria alternata*, *Monographella cucumerina*, and *Aspergillus fumigatus*, with MIC values of 0.58, 2.34, and 2.34 µg/mL, respectively, and minimum fungicidal concentration (MFC) values of 75, 75, and 37.5 µg/mL, respectively (Table 3). A dose-dependent dynamic on *Bacillus subtilis* and the fungal strains tested are presented in graphic form in Supplementary Materials (Figure S4). Lipopeptides, such as iturin and fengycin, are important antifungal agents that have been studied extensively [28]. Caspofungin is a lipopeptide antifungal agent approved for clinical use [8]. There are many more classes of CLPs which exert strong antibacterial inhibitory activity, from which daptomycin is currently under clinical use [10]. Our data show that cyanobacterial lipopeptide muscotoxins can be considered as good candidates for both structure–activity relationship studies and for development of antibiotics.

## 3. Materials and Methods

### 3.1. Chemicals and Reagents

The solvents used in HPCCC separation works were of HPLC grade and purchased from VWR (Leuven, Belgium) and Analytika (Prague, Czech Republic). The methanol used for extraction was obtained from Analytika (Prague, Czech Republic). Acetonitrile and water for HPLC-HRMS analyses were obtained either from Sigma-Aldrich (Darmstadt, Germany) or Merck (Kenilworth, NJ, USA), and were of LC-MS grade purity. Deionized water was obtained using reverse-osmosis (Ultrapur, Watrex, Prague, Czech Republic). Non-ionic polymeric adsorbents (Amberlite XAD16 and Amberlite XAD-7) were from Sigma-Aldrich (St. Louis, MO, USA).

### 3.2. Culture Growth Conditions and Biomass Extraction

The filamentous cyanobacterium *D. muscorum* CCALA 125 was cultivated in a 15 L photobioreactor on *Anabaena* medium by bubbling CO_2_-enriched air (2%) at 28 °C for 10 days [13]. The cells in the medium were harvested by centrifugation (1500*g*, 15 min), frozen at −40 °C, and then freeze-dried, affording 40 g of dried biomass. Sea sand was used for disintegration of the freeze-dried biomass and methanol (1 L) for extraction. The extraction operation was repeated three times on the same biomass. The resulting suspension (3 L) was centrifuged (5000 rpm, 10 min) and the supernatant was concentrated to total dryness using a rotary evaporator (at 40 °C). About 6200 mg of dried crude extract was obtained, which was stored at 2 °C for the subsequent enrichment process.

### 3.3. Enrichment of Crude Extract by Non-Ionic Polymeric Resins

Two non-ionic polymeric resins, that are commercially offered as XAD-16 and XAD-7 Amberlite resins, were consecutively used for enriching the dried crude extract that was obtained in the previous step. For that purpose, a suspension made of 6.200 g of dried crude extract and 500 mL of water was prepared and loaded into an Amberlite XAD-16 resin column (22 cm × 5.5 cm, 0.4 kg resin). To remove the non-adsorbed components from the column, 1 L of water was passed thorough the column. The resulting aqueous solution (1.5 L), eluted from XAD-16 resin column, was further passed through the XAD-7 resin column (22 cm × 5.5 cm, 0.4 kg resin). Again, the XAD-7 resin column was rinsed with water to remove the non-adsorbed components. Finally, the adsorbed compounds were released from each column using methanol as eluent (1.5 L). The resulting methanol eluates from each column were separately concentrated to total dryness using a rotary evaporator (at 40 °C). The dried enriched extracts were analyzed by HPLC-UV-ESI-HRMS.

### 3.4. HPCCC Separation

#### 3.4.1. HPCCC Apparatus

A HPCCC apparatus (Model Spectrum, Dynamic Extractions Ltd., Slough, UK) was used for separating the target compounds from the enriched extract. It was used a semi-preparative HPCCC column (134 mL). A H50/H150 Smart Water Chiller (LabTech Srl, Sorisole Bergamo, Italy) was used for adjusting the temperature of separation work. The stationary and mobile phases were pumped with a Q-Grad pump (LabAlliance, State College, PA, USA). The effluent from the column was continuously monitored with a Sapphire UV-VIS spectrophotometer (ECOM spol. s.r.o., Prague, Czech Republic) operating at 240 nm. The chromatographic run was processed using an EZChrom SI software platform (Agilent Technologies, Pleasanton, CA, USA).

#### 3.4.2. Selection of the Two-Phase Solvent System

Different two-phase solvent systems were prepared using *n*-hexane, ethyl acetate, ethanol, water, and acetic acid in different proportions (Table 1). The prepared solvent systems were investigated for their capacity to provide suitable partition coefficients (*K*) of the target compounds. In addition, given that the settling time as well as the density difference between the upper and lower phases of each two-phase solvent system are parameters associated with the retention of stationary phase in the column, they were thus estimated [21,26] and used as selection criteria. For estimating the partition coefficient (Table 1) of the target compound, 2 mg of extract containing the target compounds was dissolved in 2 mL of a given two-phase solvent system (1 mL per each phase). The test tube containing the sample solution was shaken vigorously, and then left, to equilibrate the two phases, for 20 min. Equal volumes of each phase were then analyzed by HPLC-ESI-HRMS to obtain the partition coefficients (*K*), which were calculated by dividing the peak area of the target compound in the upper phase by the peak area of compound in the lower phase.

#### 3.4.3. Preparation of the Two-Phase Solvent System and Sample Solution

The best two-phase solvent system selected from the previous step was prepared at a large scale for separating the target compounds by HPCCC. The components of the selected system were added into a separating funnel and shaken vigorously and were then left to equilibrate. After 20 min, the upper and lower phases were separated and degassed by applying sonication. The sample solution was prepared by dissolving the enriched extract in 3 mL of the lower phase of the selected solvent system.

#### 3.4.4. HPCCC Separation Procedure

The separation of the target compounds from the enriched extract was performed using a two-step HPCCC method. In the HPCCC first step, the solvent system composed of *n*-Hex–EtOAc–EtOH–H_2_O (1:5:1:5, *v*/*v*/*v*/*v*) was used in reverse elution mode for obtaining compounds **2**–**3**, and the **1**-containing fraction. In the HPCCC second step, the solvent system composed of *n*-Hex–EtOAc–EtOH–H_2_O–AcOH, 1:5:1:5:1 was used in reverse elution mode for obtaining the compound **1**. In the reverse elution mode, the lower phase of the two-phase solvent system is used as the mobile phase and the upper phase as the stationary phase. At the beginning, the HPCCC column was filled with the upper phase (stationary phase), and it was rotated at 1200 rpm. After that, the lower phase (mobile phase) was pumped through the column at a flow rate of 1 mL/min. When reaching the hydrodynamic equilibrium, that is, when the volume of stationary phase that is eluted from the column is constant, the sample solution was injected. The separation was performed at 28 °C. The fractions that eluted from the HPCCC column were collected and analyzed offline by HPLC-ESI-HRMS. The retention of the stationary phase (S*f*) during the HPCCC run was calculated as follows:

(1) $$Sf\;\left(\%\right)=\frac{\mathrm{Vs}}{\mathrm{Vc}}\times 100$$

where Vc is the HPCCC column volume and Vs is the volume of the stationary phase in the column at the equilibrium.

### 3.5. Subsequent Purification of HPCCC Peak Fractions by Using Semipreparative HPLC

The HPCCC peak fractions corresponding to the target compounds were repurified using a semi-preparative Agilent 1100 HPLC system. The separation was performed on a reverse phase Reprosil 100 C18 column (250 × 10 mm, 5 µm) using a mobile phase composed of acetonitrile (A) and water (B) using the following gradient: 0–2 min, 70% B; 2–6 min, 70–60% B; 6–15 min, 60–30% B; 15–16 min, 30–0% B; 16–20 min, 0–0% B; 20–21 min, 0–70% B. The peaks were detected with a diode array detector (DAD) at a monitoring wavelength of 240 nm. The mobile phase was pumped at a flow rate of 2 mL min^−1^, and the column temperature was set at 28 °C.

### 3.6. HPLC-ESI-HRMS Analysis of Extracts and HPCCC Fractions

The extracts and HPCCC fractions were analyzed using a Dionex UltiMate 3000 HPLC system (Thermo Scientific, Sunnyvale, CA, USA) coupled with a diode array detector (DAD) and with electrospray ionization sourced-high resolution mass spectrometer (ESI-HRMS; Impact HD Mass Spectrometer, Bruker, Billerica, MA, USA). The separations were performed on a reversed phase column (Phenomenex Kinetex C18 column, 150 × 4.6 mm, 2.6 μm), held at a constant temperature of 30 °C. The mobile phase of the HPLC run consisted of the combination of 0.1% formic acid in water (A) and 0.1% formic acid in acetonitrile (B). The following gradient was used: 0–1 min, 85% A; 1–20 min, 85–0% A; 20–25 min, 0% A; 25–30 min, 0–85% A, which was pumped at a constant flow rate of 0.6 mL/min. The source parameters were as follows: the spray needle voltage was set at 3.8 kV, nitrogen was used both as the nebulizing gas (3 bar) and the drying gas (12 L/min), and the temperature was 210 °C. The fragmentation of the molecular ions was induced by using nitrogen as a collision gas. The cyclic oligopeptide part of the target compounds was determined at collision energy of 60 eV. The scanning range was 50–2600 *m*/*z*, operating in the positive ion mode. The peaks were recorded with a diode array detector (DAD) at a monitoring wavelength of 240 nm.

### 3.7. Structural Identification of the Isolated Target Compounds

The chemical identity of the target compounds was determined by ESI-HRMS and NMR analysis in comparison with literature data [13]. The MS data compounds **1**–**3** are presented in Figure 5. The structure of compound **1** was determined by NMR data as presented in Figure 4 and Table 2. All NMR spectra are shown in Supplementary Materials (Figure S2). NMR spectra were recorded on a Bruker Avance III 600 MHz spectrometer equipped with TCI CryoProbe (600.23 MHz for ^1^H, 150.93 MHz for ^13^C, Bruker Biospin GmbH, Rheinstetten, Germany) in CD_3_OD, 303.2 K. The residual solvent signals were used as an internal standard (*δ*_H_ 3.330 ppm and *δ*_C_ 49.05 ppm). ^1^H-NMR, ^13^C-NMR, COSY, TOCSY, ^1^H-^13^C HSQC, ^1^H-^13^C HMBC, ^1^H-^13^C HSQC-TOCSY, and *J*-resolved spectra were measured using the standard manufacturer’s software TopSpin 3.5. The ^1^H-NMR spectrum was zero-filled to 2-fold data points and multiplied by a window function (two parameter double-exponential Lorentz-Gauss function), before Fourier transformation to improve the resolution. The ^13^C-NMR spectrum was zero filled to 2-fold data points. Subsequently, the line broadening (1 Hz) was used to improve signal-to-noise ratio. Protons were assigned by COSY, TOCSY, and HSQC-TOCSY, and the assignment was transferred to carbons by HSQC. The chemical shifts are given on the *δ* scale (ppm), and coupling constants are given in Hz. The digital resolution allowed us to present the proton and carbon chemical shifts to three or two decimal places. The proton chemical shift readouts from HSQC are reported to two decimal places. The operating conditions of the ESI-HRMS instrument are indicated in the previous section.

### 3.8. In Vitro Antimicrobial Bioassay

Due to the unavailability of enough amounts of isolated compounds, the antifungal activity for all the isolated compounds (**1**–**3**) was initially tested only against plant pathogenic fungi *Sclerotinia sclerotiorum* by determining the minimum inhibitory concentration (MIC) using disc diffusion assay. Compounds (**1**–**3**) were dissolved in MeOH at different concentrations and further transferred (20 µL) on layer filter paper targets (size 5 mm) to get desired concentrations of 250–1.5625 µg/disk. Discs were placed at the border of Petri dish (average 35 mm) containing Czapek-Dox medium. The mycelial target of the plant pathogenic fungus *S. sclerotiorum* (0.5 cm in diameter) was inoculated on the opposite sites of dish. The fungicide RANCONA 15 ME (active substance—ipconazole, Crompton Registration Ltd., London, UK) and methanol were used as positive and negative control, respectively. Each experiment was performed in triplicate. The growth of fungal mycelium after 4 days in the presence of the studied compounds was evaluated using digital image analysis. The macro-optic system, consisting of a digital camera DUS-1 (5Mpx) and objective lens type Cosmicar (Nikon Instruments, CS-Optoteam Ltd., Prague, Czech Republic) were used for acquisition of images of the Petri dishes. The images (TIFF format) were converted by thresholds, and the area of binary objects corresponding to size of grown mycelium was measured and evaluated by software NIS-ELEMENTS AR ver. 3.2 (LIM—Laboratory Imaging Ltd., Prague, Czech Republic) and by a method published previously [29]. The inhibition of the mycelium growth (inhibition zone in percentages) was calculated as the difference between the measurements (in px) of the total area of fungal mycelium (negative controls) and of the area that is covered with mycelium (treated dishes).

Furthermore, compound **1** was evaluated for its antimicrobial activity against five bacterial isolates; *S. aureus* (CCM3824), *B. subtilis* (CCM1999), *S. sanguinis* (CCM4047), *P. aeruginosa* (CCM1959), *E. coli* (CCM2024), and eight fungal isolates; *C. friedrichii* (BCC020_2879), *A. fumigatus* (BCC020_2845), *F. oxysporum* (BCC020_2866), *T. harzianum* (BCC020_0606), *B. sorokiniana* (BCC020_1571), *M. cucumerina* (BCC020_2872), *C. globosum* (BCC020_2527), and *A. alternata* (BCC020_0609) using broth two-fold microdilution method following the standard protocol [30]. Briefly, for broth microdilution method, the compounds were serially diluted at varying concentrations between 300 and 0.0732 µg/mL in Mueller Hinton broth (for bacteria) and Muller Hinton broth with 2% glucose (for fungi) in 96-well plates. Culture broth (0.1 mL) was added to each well containing the compound. Plates were incubated aerobically at 37 °C for 16 h (bacteria) and 30 °C for 48 h (for fungi). Negative controls were prepared separately with respective organisms in the same culture media with methanol (the dissolving solvent). Positive controls were also prepared separately with respective organisms. Erythromycin (32–0.0625 µg/mL) was used as positive control for *S. aureus* and *S. sanguinis*, gentamycin (32–0.0625 µg/mL) was used as positive control for *Bacillus subtilis*, *E. coli*, and *P. aeruginosa*, whereas fluconazole (32–0.0625 µg/mL) was used as positive control for all the fungal isolates. After incubation, the well with the least concentration of the compound showing any inhibition in the growth was taken as the MIC value for the respective organisms. MIC_95_ was determined as the concentration required to inhibit or kill 95% of bacterial isolates spectroscopically at 600 nm. Minimum fungicidal concentration (MFCs) were determined after 48 h incubation by removing 10 µL of the contents from wells showing no visible growth and spreading them on to Sabouraud dextrose agar plates [31]. The plates were then incubated for 72 h and MFCs were determined as the concentrations which killed 95–100% of the inoculum. All the experiments were done in triplicate.

## 4. Conclusions

Three CLPs (muscotoxins A–C) were isolated and purified from the filamentous cyanobacterium *Desmonostoc muscorum strain CCALA125* by the combined use of adsorption on polymeric resins, HPCCC, and HPLC methods. As these three chromatographic methods are recognized to be orthogonal against each other, therefore, their combined use rendered the separation of chemically similar compounds. Muscotoxin C was isolated for the first time and its chemical structure was established by MS and NMR spectroscopic data. The described isolation method is an efficient approach for obtaining CPLs from cyanobacterial biomass and represents a methodological reference than can be scaled up for obtaining these compounds in higher amounts. Muscotoxin A was found to exhibit a potent antifungal activity against plant pathogenic fungi, thus, it might have applications as an agricultural fungicide or be used as a potential chemical template for developing new fungicides.

## Figures and Tables

**Table 1:** The partition coefficient (*K*) and separation factor (α) values of three muscotoxins from *D. muscorum* CCALA 125 in different two-phase solvent systems and the settling times.

Solvent Systems	Composition	Relative Proportions of Solvents (*v*/*v*/*v*/*v*)	Phase Volume Ratio (UP/LP)	Settling Time (s)	Density Difference (LP-UP, g/mL)	Partition Coefficient (*K*) of Muscotoxins		
						1	2	3
1	*n*-Hex–EtOH–H_2_O	10:5:5	1.22	7	0.272	0.00	0.03	0.01
2	*n*-Hex–EtOAc–EtOH–H_2_O	9:1:5:5	1.00	10	0.226	0.00	0.03	0.01
3	*n*-Hex–EtOAc–EtOH–H_2_O	8:2:5:5	1.00	11	0.197	0.00	0.01	0.00
4	*n*-Hex–EtOAc–EtOH–H_2_O	7:3:5:5	0.85	14	0.229	0.01	0.06	0.02
5	*n*-Hex–EtOAc–EtOH–H_2_O	6:4:5:5	0.82	18	0.197	0.01	0.09	0.04
6	*n*-Hex–EtOAc–EtOH–H_2_O	5:5:5:5	0.82	33	0.183	0.00	0.01	0.01
7	*n*-Hex–EtOAc–EtOH–H_2_O	4:5:4:5	1.00	33	0.133	0.02	0.14	0.05
8	*n*-Hex–EtOAc–EtOH–H_2_O	3:5:3:5	1.00	21	0.152	0.08	0.32	0.27
9	*n*-Hex–EtOAc–EtOH–H_2_O	2:5:2:5	1.12	18	0.139	0.40	0.97	1.22
10	*n*-Hex–EtOAc–EtOH–H_2_O	1:5:1:5	1.00	13	0.112	0.36	0.94	1.41
11	*n*-Hex–EtOAc–EtOH–H_2_O–AcOH	1:5:1:5:1	0.97	24	0.119	1.09	1.97	2.45

*n*-Hex: *n*-hexane. EtOAc: ethyl acetate. EtOH: ethanol. AcOH: acetic acid.

**Table 2:** ^1^H and ^13^C-NMR data of **1** (600.13 MHz for ^1^H, 150.93 MHz for ^13^C, CD3OD, 303.2 K).

10	Atom	*δ*_C_ [ppm]	*m*	*δ*_H_ [ppm]	*n*_H_	*m*	*J*_HH_ [Hz]
**X^1^**	1	174.68	s	-	0	-	-
	2	73.12	d	4.166	1	d	2.2
	3	51.09	d	4.330	1	m	-
	4	40.57	t	1.82^H^	1	m	-
				1.66^H^	1	m	-
	5	69.67	d	3.61^H^	1	m	-
	6	38.16	t	1.53^H^	1	m	-
				1.42^H^	1	m	-
	7	26.41	t	1.50^H^	1	m	-
				1.35^H^	1	m	-
	8	33.10	t	1.31^H^	2	m	-
	9	23.76	t	1.34^H^	2	m	-
	10	14.44	q	0.904	3	m	-
**Gln^2^**	C=O	173.76	s	-	0	-	-
	α	54.24	d	4.479	1	m	-
	β	29.02	t	2.24^H^	1	m	-
				1.96^H^	1	m	-
	γ	32.96	t	2.298	1	m	-
				2.24^H^	1	m	-
	δ	178.48	s	-	0	-	-
**Gly^3^**	C=O	170.70	s	-	0	-	-
	α	43.46	t	4.118	1	d	17.3
				3.951	1	d	17.3
**Pro^4^**	C=O	174.59	s	-	0	-	-
	α	62.33	d	4.289	1	m	-
	β	30.55	t	2.12^H^	1	m	-
				1.65^H^	1	m	-
	γ	25.56	t	1.87^H^	1	m	-
				1.66^H^	1	m	-
	δ	48.01	t	3.62^H^	1	m	-
				3.53^H^	1	m	-
**Phe^5^**	C=O	173.79	s	-	0	-	-
	α	56.33	d	4.62^H^	1	m	-
	β	37.42	t	3.29^H^	1	m	-
				2.952	1	dd	10.3, 14.0
	*ipso*	138.87	s	-	0	-	-
	*ortho*	130.15	d	7.231	2	m	-
	*meta*	129.69	d	7.288	2	m	-
	*para*	127.94	d	7.209	1	m	-
**Val^6^**	C=O	174.83	s	-	0	-	-
	α	61.02	d	4.139	1	d	7.8
	β	30.93	d	2.21^H^	1	m	-
	γ	19.10	q	0.89^H^	3	m	-
	β-Me	19.80	q	0.842	3	d	6.7
**Ser^7^**	C=O	171.89	s	-	0	-	-
	α	57.97	d	4.43^H^	1	m	-
	β	62.80	t	3.922	1	dd	5.2, 11.3
				3.882	1	dd	4.9, 11.3
**dThr^8^**	C=O	167.07	s	-	0	-	-
	α	130.78	s	-	0	-	-
	β	127.80	d	5.912	1	q	7.4
	γ	13.68	q	1.921	3	d	7.4
**Ser^9^**	C=O	172.40	s	-	0	-	-
	α	57.14	d	4.610	1	m	-
	β	63.45	t	4.000	1	dd	5.3, 11.5
				3.797	1	dd	4.1, 11.5
**Ile^10^**	C=O	172.25	s	-	0	-	-
	α	56.53	d	4.640	1	d	7.2
	β	38.44	d	1.93^H^	1	m	-
	γ	27.42	t	1.42^H^	1	m	-
				1.170	1	m	-
	δ	12.13	q	0.948	3	t	7.3
	β-Me	15.12	q	0.928	3	d	6.8
**Pro^11^**	C=O	173.53	s	-	0	-	-
	α	61.69	d	4.408	1	m	-
	β	30.48	t	2.04^H^	2	m	-
	γ	25.56	t	1.99^H^	2	m	-
	δ	48.9^H^	t	3.83^H^	1	m	-
				3.62^H^	1	m	-

**Table 3:** Antimicrobial inhibitory effect of muscotoxin A (**2**) against Gram-positive and Gram-negative bacteria and plant pathogenic fungi.

Tested Microorganisms	(µg/mL)	
**Bacteria**	**MIC**	**MIC_95_**
*Staphylococcus aureus*	NA	ND
*Bacillus subtilis*	37.5 (4)	≤300
*Streptococcus sanguinis*	NA	ND
*Pseudomonas aeroginosa*	NA	ND
*Escherichia coli*	NA	ND
**Fungi**		**MFC**
*Candida friedrichii*	75 (8)	<300
*Aspergillus fumigatus*	2.34 (32)	37.5
*Fusarium oxysporum*	75 (8)	<300
*Trichoderma harzianum*	37.5 (4)	300
*Bipolaris sorokiniana*	NA	ND
*Monographella cucumerina*	2.34 (2)	75
*Chaetomium globosum*	18.75 (2)	<300
*Alternaria alternata*	0.58 (2)	75

The MIC value for the standard antibiotic used as positive control (µg/mL) are given within parenthesis. NA—no activity; ND—not determined.

## Supplementary Materials

The following are available online. Figure S1: The HPLC-ESI-HRMS chromatograms of separated muscotoxins. Figure S2: 1D, 2D NMR experiments of compound **1**. Figure S3: In vitro antifungal bioassay against plant pathogenic fungi *S. sclerotiorum*. Figure S4: Dose dependent inhibition profile and determination of minimum inhibitory concentration (MIC) of muscotoxin (**2**) against *Bacillus subtilis* and seven fungal isolates tested.
