# Supplementary material for: Application of HPCCC Combined with Polymeric Resins and HPLC for the Separation of Cyclic Lipopeptides Muscotoxins A–C and Their Antimicrobial Activity
Source: Molecules. 2018 Oct 16;23(10):2653. doi: 10.3390/molecules23102653 (PMC6222847; doi:10.3390/molecules23102653)
Supplement: Supplementary file 1 [file molecules-23-02653-s001.zip › molecules-338441-supplementary/molecules-338441-supplementary-Figure S4.pdf]

Figure S4: Dose dependent inhibition profile and determination of minimum inhibitory concentration (MIC) of Muscotoxin (2) against *Bacillus subtilis* and seven fungal isolates tested.

Application of HPCCC combined with polymeric resins and HPLC for the separation of cyclic lipopeptides muscotoxins A-C and their anti-microbial activity.

José Cheel [1*], Jan Hájek [1], Marek Kuzma[2], Kumar Saurav [1], Iva Smýkalová[3], Eliška Ondráčková[3], Petra Urajová [1], Dai Long Vu [1], Karine Faure [4], Jiří Kopecký [1] and Pavel Hrouzek [1*]

1 Laboratory of Algal Biotechnology-Centre ALGATECH, Institute of Microbiology of the Czech Academy of Sciences, Opatovický mlýn, Novohradská 237, 379 81, Třeboň, Czech Republic; jcheel@alga.cz (J.C.) ; hajek@alga.cz (J.H); urajova@alga.cz (P.U.); longvu@alga.cz (D.L.V.); sauravverma17@gmail.com (K.S.); kopecky@alga.cz (J.K.); hrouzek@alga.cz (P.H.)
2 Laboratory of Molecular Structure Characterization, Institute of Microbiology of the Czech Academy of Sciences, Vídeňská 1083, 142 20 Prague, Czech Republic; kuzma@biomed.cas.cz
3 Plant Biotechnology Department, AGRITEC Plant Research Ltd., Zemědělská 2520/16, 787 01 Šumperk, Czech Republic; smykalova@agritec.cz; ondrackova@agritec.cz
4 University of Lyon, CNRS, Université Claude Bernard Lyon 1, Ens de Lyon, Institut des Sciences Analytiques, UMR 5280, 5 rue de la Doua, 69100, Villeurbanne, France; karine.faure@isa-lyon.fr

* Correspondence: jcheel@alga.cz; jcheel@email.cz (J.C.); hrouzek@alga.cz (P.H.) ; Tel.: +420-384-340-465

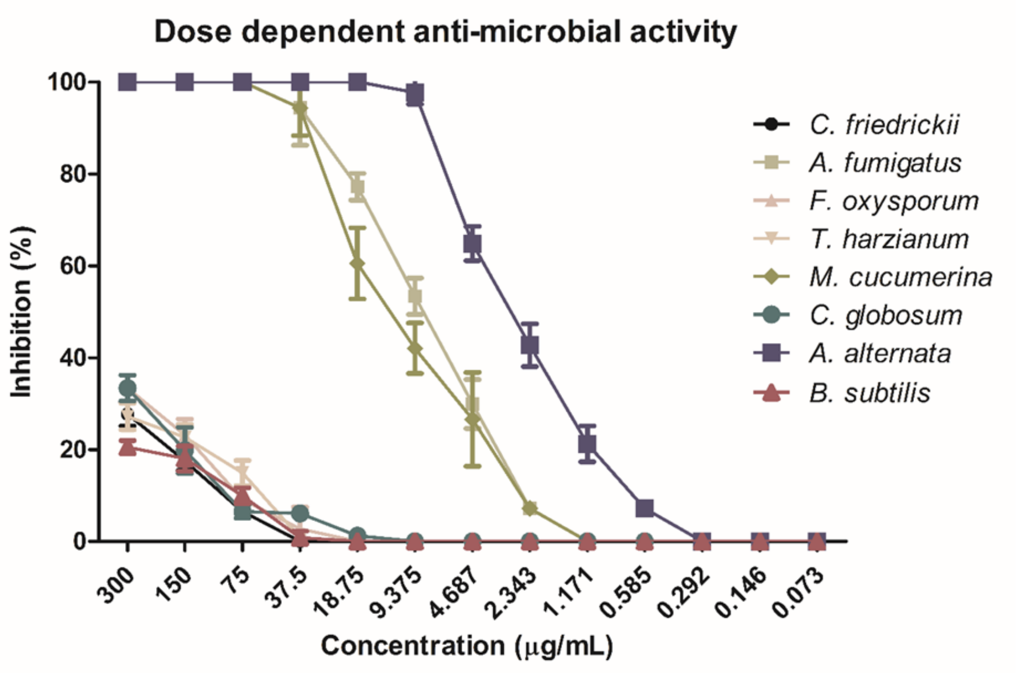

Figure S4: Dose dependent inhibition profile and determination of minimum inhibitory concentration (MIC) of Muscotoxin A (**2**) against *Bacillus subtilis* and seven fungal isolates tested. Inhibition percentage (%) was calculated using the control experiment representing the growth of each tested organism grown in the presence of the diluting solvent used (methanol).
